# Outcome of ERCP related to case-volume

**DOI:** 10.1007/s00464-021-08915-y

**Published:** 2022-01-03

**Authors:** Eva-Lena Syrén, Gabriel Sandblom, Lars Enochsson, Arne Eklund, Bengt Isaksson, Johanna Österberg, Staffan Eriksson

**Affiliations:** 1 Department of Surgical Sciences, Uppsala University, 751 35 Uppsala, Sweden; 2 Centre for Clinical Research, Uppsala University, Region Västmanland, Uppsala, Sweden; 3 Department of Surgery, Hospital of Västmanland, Västerås, Sweden; 4 Department of Clinical Science and Education Södersjukhuset, Karolinska Institute, Stockholm, Sweden; 5 Department of Surgical and Perioperative Sciences, Surgery, Umeå University, Umeå, Sweden; 6 Department of Surgery, Mora Hospital, Mora, Sweden; 7 Department of Surgery, Akademiska Hospital, Uppsala, Sweden; 8 Department of Surgery, Södersjukhuset, Stockholm, Sweden; 9 Department of Clinical Sciences, Intervention and Technology (CLINTEC), Karolinska Institute, Stockholm, Sweden

**Keywords:** ERCP, Case-volume, Cannulation rate, Procedure time, Intra- and postoperative complication rates, Post-ERCP pancreatitis

## Abstract

**Background and aims:**

In some studies, high endoscopic retrograde cholangiopancreatography (ERCP) case-volume has been shown to correlate to high success rate in terms of successful cannulation and fewer adverse events. The aim of this study was to analyze the association between ERCP success and complications, and endoscopist and centre case-volumes.

**Methods:**

Data were obtained from the Swedish National Register for Gallstone Surgery and ERCP (GallRiks) on all ERCPs performed for Common Bile Duct Stone (CBDS) (*n* = 17,873) and suspected or confirmed malignancy (*n* = 6152) between 2009 and 2018. Successful cannulation rate, procedure time, intra- and postoperative complication rates and post-ERCP pancreatitis (PEP) rate, were compared with endoscopist and centre ERCP case-volumes during the year preceding the procedure as predictor.

**Results:**

In multivariable analyses of the CBDS group adjusting for age, gender and year, a high endoscopist case-volume was associated with higher successful cannulation rate, lower complication and PEP rates, and shorter procedure time (*p* < 0.05). Centres with a high annual case-volume were associated with high successful cannulation rate and shorter procedure time (*p* < 0.05), but not lower complication and PEP rates.

When indication for ERCP was malignancy, a high endoscopist case-volume was associated with high successful cannulation rate and low PEP rates (*p* < 0.05), but not shorter procedure time or low complication rate. Centres with high case-volume were associated with high successful cannulation rate and low complication and PEP rates (*p* < 0.05), but not shorter procedure time.

**Conclusions:**

The results suggest that higher endoscopist and centre case-volumes are associated with safer ERCP and successful outcome.

Endoscopic retrograde cholangiopancreatography (ERCP) is the standard procedure to diagnose and treat conditions in the biliary and pancreatic ducts such as common bile duct stone (CBDS) and biliary tract malignancy. In unselected population-based settings, successful cannulation is achieved in > 85% of cases [1, 2]. The complexity of ERCP, however, ranges from uncomplicated extraction of small stones to extremely challenging procedures such as hilar stenting, electrohydraulic lithotripsy (EHL) for difficult stones, and oral cholangioscopy or pancreatoscopy. ERCP complexity can be graded according to Schutz’s criteria [3] or the Cotton classification [4]. The Cotton scale includes not only the complexity of the endoscopic procedure but also the experience of the endoscopist.

Existing complexity grading scales lack validation, and to be able to compare results from different endoscopic centres, and thereby allocate resources, a new ERCP complexity grading scale, the H.O.U.S.E. classification was designed and developed at the Karolinska University Hospital Huddinge in 2017. H.O.U.S.E. includes three ERCP categories: Category I, uncomplicated ERCP; Category II, ERCP of intermediate complexity: and Category III, highly complicated ERCP. The H.O.U.S.E. classification was shown to predict procedure time and to some extent adverse events [5].

Several complications are associated with ERCP the most common being post-ERCP pancreatitis (PEP) with a rate of 3.5–5% [1, 6–8]. The risk for developing PEP is correlated to technical factors, complexity of the procedure, and patient-related variables [7–13]. Although PEP is widely accepted as the primary adverse outcome measure following ERCP, the risk factors for PEP also are associated with other adverse events such as bleeding, perforation, and other procedure-related complications. PEP may thus be considered a surrogate endpoint for safety and success of ERCP.

Lack of experience has been shown to be associated with poor outcome in major surgical procedures [14]. Likewise, larger ERCP case-volumes are associated with higher success rates in terms of successful cannulation and fewer complications [15–21]. Studies have shown that high-volume ERCP centres have better results and lower complication rates than low-volume centres [17, 18, 22, 23]. However, there are also data showing that low-volume units can also perform safe ERCPs [24–26]. It is difficult to say whether these conflicting results depend on the experience of the endoscopist or routines at the centres where the ERCPs are performed. Centralization of complex ERCPs to high-volume centres with highly experienced endoscopists may well increase the safety and success of this procedure. Population-based studies are needed to confirm this hypothesis.

The aim of this study was to compare highly and less experienced endoscopists as well as high and low-volume centres, regarding successful cannulation rates, procedure times, intraoperative complication rates, and postoperative complications rates within 30 days (PEP, perforation and intra- and postoperative bleeding), of ERCPs performed for common bile duct stone or malignancy.

## Materials and methods

This study is based on data retrieved from the Swedish National Register for Gallstone Surgery and ERCP, GallRiks, which was created 2005 under direction of the Swedish National Board of Health and Welfare and the Swedish Surgical Society and administered by the Uppsala Clinical Research Center (UCR). GallRiks covers about 90% of cholecystectomies and ERCPs performed in Sweden, and practically all Swedish hospitals participate. Most of these procedures are performed by surgeons, even if gastroenterologists are responsible for a smaller proportion of ERCPs. Patient- and procedure-related data as well as intraoperative complications and postoperative complications within 30 days are prospectively registered. The completeness of 30-day follow-up is approximately 95%. GallRiks is regularly validated, and the validation process and the results of national coverage are published each year [1, 27–29]. Consent from the patient to participate in register-based research is required for registration in GallRiks. Patients are able to withdraw their personal data from the register at any time. PEP was defined as: (1) typical abdominal pain; (2) serum amylase elevation > 3 times the upper limit longer than 24 h after ERCP; and (3) need for hospitalization according to the Cotton criteria [7].

**Fig. 1:**
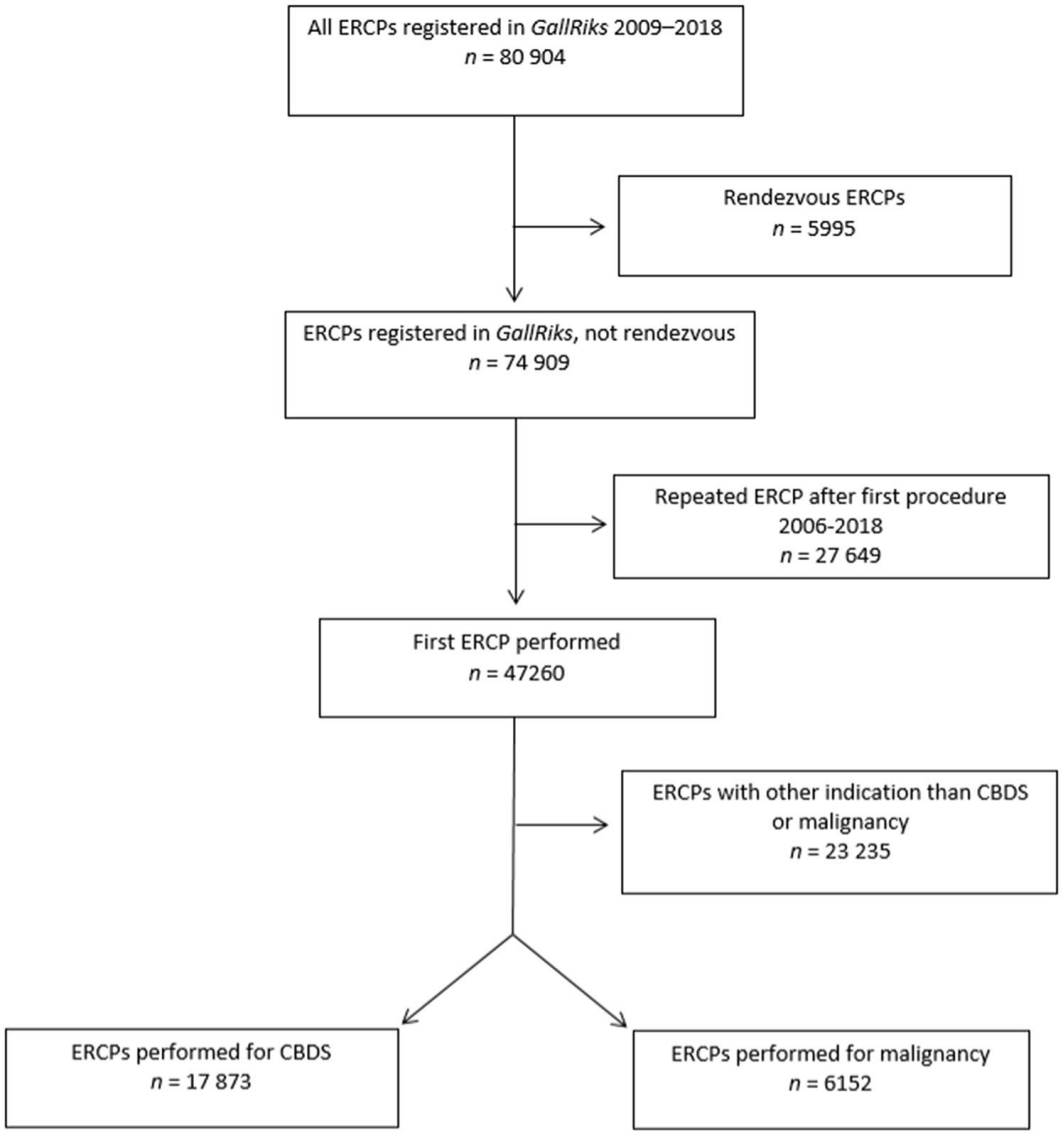
Flow chart showing study group assembly

Data from GallRiks on all ERCPs 2009–2018 performed for common bile duct stone (*n* = 17,873) and malignancy (*n* = 6152), with complete registration and 30-day follow-up, were collected and compiled. Procedures for any other indication, procedures on patients having undergone previous ERCP since 2006, and rendezvous ERCPs were excluded from the analysis (Fig. 1). Associations between both endoscopist ERCP case-volume and centre volume, and successful cannulation rate, procedure time, intraoperative complication rate, and postoperative complication rate within 30 days (PEP, perforation, and intra- and postoperative bleeding) were analyzed. Volumes were based on those during the year preceding the observations. When calculating cumulative volume of ERCP procedures for endoscopists and centers no ERCPs were excluded.

The Regional Ethics Review Board in Stockholm approved the study 17th June 2020 (IRB-approval, reference number: 2020-01450).

Consent from the patient to participate in register-based research is required for registration in GallRiks. Patients are given the opportunity to withdraw all their personal data at any time from the register.

## Statistics

**Fig. 2:**
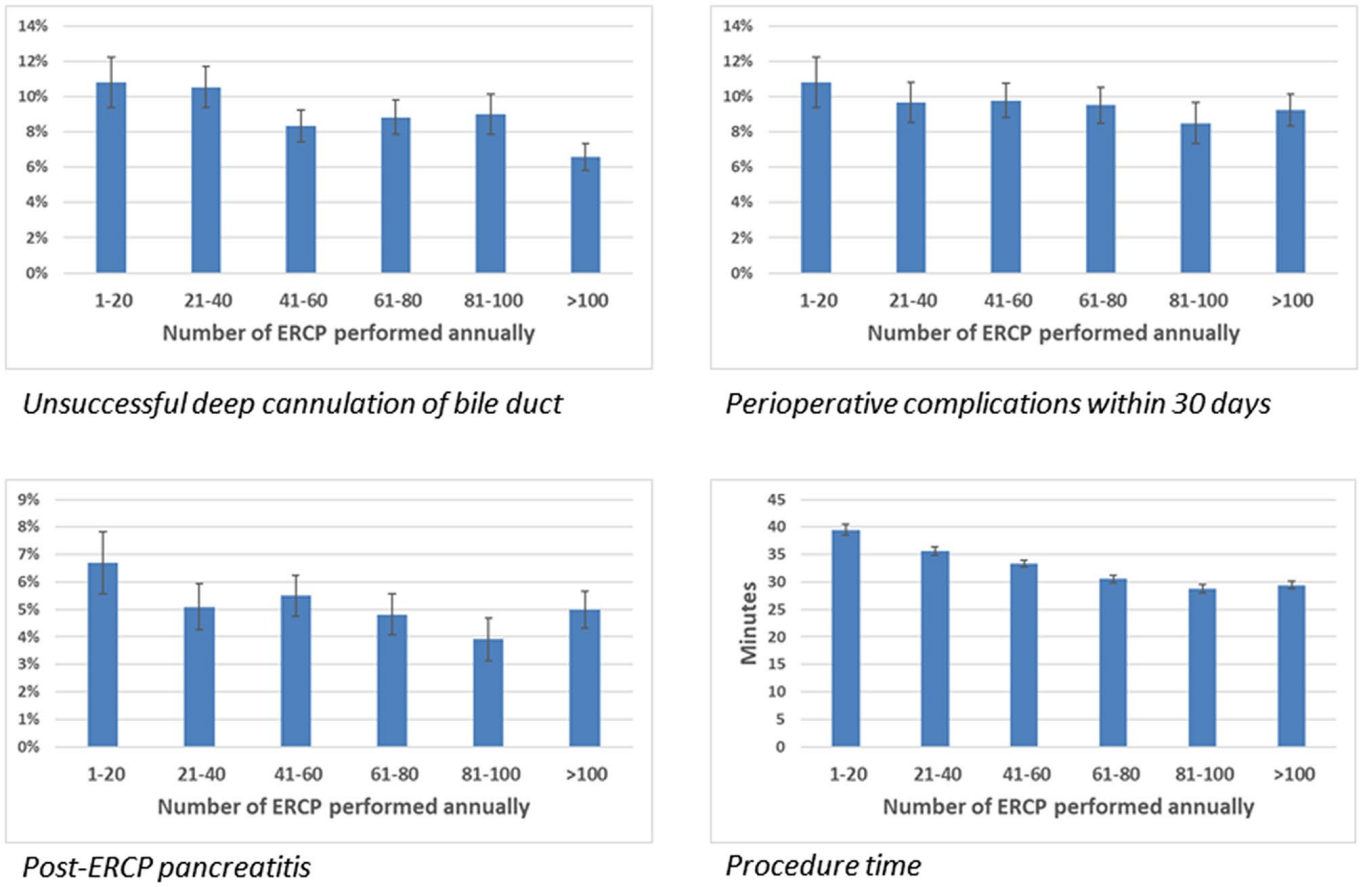
ERCPs 2009–2018 with indication common bile duct stone. Univariable and multivariable logistic regression analyses of ERCP volumes (endoscopist) during the year preceding the procedure with successful deep cannulation of bile duct (in this figure illustrated as unsuccessful deep cannulation), intra- and postoperative complications within 30 days and post-ERCP pancreatitis (PEP) as outcome. Univariable and multivariable linear regression analyses of ERCP volumes (endoscopist) during the year preceding the procedure with procedure duration as outcome

Univariable and multivariable logistic regression analyses with the endpoints successful cannulation, procedure time, intraoperative complication rate, and postoperative complication rate within 30 days (PEP, perforation, and intra- and postoperative bleeding) were performed with endoscopist and centre volumes as the variables. In the multivariable logistic regression analyses, adjustments were made for age, gender, and year of ERCP. The adjustments made in the multivariable analysis were based on assumptions of cause–effect relationships. Analyses were made with volumes on log scales (*n* = 0–4, 5–10, 11–20, 21–40, 41–80, 81–160 or 161–320 for endoscopist and *n* = 0–20, 21–40, 41–80, 81–160, 161–320 or > 320 for centre). In Fig. 2 volumes are presented as an arithmetic scale.

## Results

ERCP for CBDS was more common in women (58.7%). Mean age of patients undergoing ERCP for CBDS was 67.1 years. ERCP for malignancy was more equally distributed between the sexes, mean age being 71.6 years. The proportion of procedures performed by an endoscopist with an ERCP case-volume > 80 the preceding year increased from 37% in 2009 to 40% in 2018. The proportion of procedures performed at a centre with an ERCP volume > 160 the preceding year increased from 70% in 2009 to 78% in 2018 (Table 1). Regarding degrees of complexity of ERCPs performed by endoscopists and at centres with different procedure volumes, no major changes occurred during the study period. Procedures classified as H.O.U.S.E. II or III were performed at centres with a procedure volume > 160 in 71% (*n* = 1179) in 2009 and 83% (*n* = 1493) in 2018. The percentage of procedures classified as H.O.U.S.E. II or III performed by endoscopists with an ERCP case-volume > 80 increased from 41% (*n* = 689) in 2009 to 47% (*n* = 851) in 2018.

**Table 1:** Baseline characteristics of the cohort 2009–2018

	ERCP for common bile duct stones (*N* = 17,873)	ERCP for malignancy (*N* = 6152)
Gender		
Men	7373 (41.3%)	2944 (47.9%)
Women	10,492 (58.7%)	3206 (52.1%)
Unknown	8 (< 0.01%)	2 (< 0.01%)
Mean age, years	67.1 (y)	71.6 (y)
Year of ERCP		
2009	1260 (7.0%)	538 (8.7%)
2010	1786 (10.0%)	497 (8.1%)
2011	1872 (10.5%)	515 (8.4%)
2012	1757 (9.8%)	559 (9.1%)
2013	1799 (10.1%)	613 (10.0%)
2014	1905 (10.7%)	583 (9.5%)
2015	1905 (10.7%)	652 (10.6%)
2016	1924 (10.8%)	783 (12.7%)
2017	1881 (10.5%)	669 (10.9%)
2018	1784 (10.0%)	743 (12.1%)
Number of ERCPs performed by endoscopist previous year		
0–5	467 (2.6%)	109 (1.8%)
6–10	423 (2.4%)	98 (1.6%)
11–20	1111 (6.2%)	255 (4.1%)
21–40	2726 (15.3%)	816 (13.3%)
41–80	6884 (38.5%)	2230 (36.2%)
81–160	5483 (30.7%)	2247 (36.5%)
161–320	779 (4.4%)	397 (6.5%)
Number of ERCPs performed at centre previous year		
0–5	50 (0.3%)	8 (0.1%)
6–10	76 (0.4%)	6 (0.1%)
11–20	215 (1.2%)	34 (0.6%)
21–40	410 (2.3%)	97 (1.6%)
41–80	1368 (7.7%)	418 (6.8%)
81–160	3398 (19.0%)	1050 (17.1%)
161–320	8098 (45.3%)	2712 (44.1%)
> 320	4258 (23.8)	1827 (29.7%)

Regarding ERCP for CBDS, higher endoscopist ERCP case-volume as well as centre volume were correlated to higher rate of successful deep cannulation of the bile duct, shorter procedure time, lower intraoperative complication rate, lower postoperative complication rate within 30 days, and lower PEP rate. In the multivariable analysis gender was not significant when it came to procedure time (Table 2, Fig. 2).

**Table 2:** ERCPs 2009–2018 with indication common bile duct ston

Endoscopist case-volume					Centre case-volume				
Outcome	Univariable		Multivariable*		Outcome	Univariable		Multivariable*	
	Odds ratio (95% CI)	*p*	Odds ratio (95% CI)	*p*		Odds ratio (95% CI)	*p*	Odds ratio (95% CI)	*p*
Successful deep cannulation of bile duct									
Endoscopist annual ERCP volume	1.187 (1.172–1.202)	< 0.001	1.093 (1.078–1.108)	< 0.001	Centre annual ERCP volume	1.083 (1.037–1.131)	< 0.001	1.084 (1.038–1.133)	< 0.001
Intra- and postoperative complications within 30 days									
Endoscopist annual ERCP volume	0.951 (0.913–0.990)	0.015	0.950 (0.912–0.989)	0.013	Centre annual ERCP volume	1.007 (0.962–1.053)	0.775	1.006 (0.961–1.053)	0.794
Post-ERCP pancreatitis									
Endoscopist annual ERCP volume	1.044 (1.018–1.070)	< 0.001	1.028 (1.002–1.054)	0.034	Centre annual ERCP volume	0.953 (0.901–1.009)	0.099	0.954 (0.902–1.010)	0.103

Procedure duration (minutes)									
	Standardized coefficient beta	*p*	Standardized coefficient beta	*p*		Standardized coefficient beta	*p*	Standardized coefficient beta	*p*
Endoscopist annual ERCP volume	− 2.574 (− 2.824 to − 2.323))	< 0.001	− 2.579 (− 2.828 to − 2.330)	< 0.001	Centre annual ERCP volume	− 2.523 (− 2.796 to − 2.250)	< 0.001	− 2.583 (− 2.855 to − 2.310)	< 0.001

Univariable and multivariable logistic regression analyses of ERCP volumes (endoscopist and centre) during the year preceding the procedure with successful deep cannulation of bile duct, intra- and postoperative complications within 30 days and post-ERCP pancreatitis (PEP) as outcomes. Univariable and multivariable linear regression analyses of ERCP volumes (endoscopist and centre) during the year preceding the procedure with procedure duration as outcome

*Adjusted for age, gender and year of ERCP

Regarding ERCP for malignancy, results were not as clear as for ERCP performed for CBDS. Higher endoscopist volume and centre volume correlated with a higher rate of successful deep cannulation of the bile duct, but not to shorter procedure time. Intraoperative complication rate, postoperative complication rate within 30 days, and PEP rate were lower at high-volume centres but endoscopist case-volume showed no correlation (Table 3).

**Table 3:** ERCPs 2009–2018 with indication malignancy

Endoscopist case-volume					Centre case-volume				
Outcome	Univariable		Multivariable*		Outcome	Univariable		Multivariable*	
	Odds ratio (95% CI)	*p*	Odds ratio (95% CI)	*p*		Odds ratio (95% CI)	*p*	Odds ratio (95% CI)	*p*
Successful deep cannulation of bile duct									
Endoscopist annual ERCP volume	1.158 (1.100–1.218)	< 0.001	1.155 (1.097–1.216)	< 0.001	Centre annual ERCP volume	1.153 (1.088–1.222)	< 0.001	1.143 (1.078–1.212)	< 0.001
Intra- and postoperative complications within 30 days									
Endoscopist annual ERCP volume	1.068 (0.984–1.159)	0.118	1.062 (0.978–1.153)	0.151	Centre annual ERCP volume	1.206 (1.092–1.331)	< 0.001	1.186 (1.074–1.309)	0.001
Post-ERCP pancreatitis									
Endoscopist annual ERCP volume	1.190 (1.056–1.341)	0.004	1.179 (1.045–1.330)	0.008	Centre annual ERCP volume	1.425 (1.230–1.651)	< 0.001	1.362 (1.174–1.579)	< 0.001

Procedure duration (minutes)									
	Standardized coefficient beta	*p*	Standardized coefficient beta	*p*		Standardized coefficient beta	*p*	Standardized coefficient beta	*p*
Endoscopist annual ERCP volume	− 0 .207 (− 0.768 to 0.354)	0.470	− 0.288 (− 0.848 to 0.271)	0.312	Centre annual ERCP volume	− 0.365 (− 1 .000 to 0.270)	0.260	− 0.637 (− 1.274 to − 0.001)	0.050

Univariable and multivariable logistic regression analyses of ERCP volumes (endoscopist and centre) during the year preceding the procedure with successful deep cannulation of bile duct, intra- and postoperative complications within 30 days and post-ERCP pancreatitis (PEP) as outcomes. Univariable and multivariable linear regression analyses of ERCP volumes (endoscopist and centre) during the year preceding the procedure with procedure duration as outcome

*Adjusted for age, gender and year of ERCP

## Discussion

In this study, based on prospectively retrieved data over a period of 10 years, the association between ERCP case-volume, both endoscopist and centre, and successful cannulation, procedure time and adverse events, were analyzed. The relationship between larger ERCP case-volumes and higher success rates has been described in previous studies [20, 23]. In this study, however, we chose to focus on two clearly defined indications for ERCP: common bile duct stone (CBDS) and malignancy. ERCPs for common bile duct stone in Sweden are performed in many hospitals of varying size and capacity and by endoscopists with different experience. ERCP procedures for malignancy, on the other hand, are often more complex and therefor often performed by endoscopists with greater experience of advanced endoscopy [1, 27, 28].

The validity could have been improved if the study had been based on a larger number of ERCP procedures. To get a well-defined study-population and to minimize potential sources of error we, however, decided to exclude ERCPs for other indications than choledocholithiasis and malignancy. During the study period 2009–2018 many ERCPs were registered in the Swedish National Register for Gallstone Surgery and ERCP (GallRiks) as having been performed on indication jaundice. This symptom is commonly seen in patients with CBDS as well as in patients with malignancy of the pancreas or the biliary ducts. If we had included these procedures, it would have been very difficult to draw any certain conclusions of differences in outcomes between the two study groups. Since 2021 the choice of jaundice as indication for ERCP has been removed from GallRiks in order to avoid misunderstandings about which condition necessitated the procedure.

Since several years the most common management of CBDS detected by cholangiography during cholecystectomy in Sweden is intraoperative rendezvous ERCP [1, 30]. In these cases, access to the bile duct is facilitated by an antegrade guidewire from the cystic duct to the duodenum, and the rate of unsuccessful perioperative complications, particularly PEP, is low. Since we aimed at including only patients with an untouched major duodenal papilla, to properly assess the parameters cannulation success and PEP-rate, we had to exclude the relatively large group of rendezvous ERCPs [31, 32].

Non-rendezvous ERCPs performed for CBDS may be complicated; large impacted stones, for example, that require advanced methods such as electrohydraulic lithotripsy (EHL). The majority of ERCPs for CBDS, however, are uncomplicated and fall into the H.O.U.S.E. category I [5] or Cotton and Schutz Grade II [3, 4]. Endoscopists with the greatest experience and centres with the highest volumes had the highest cannulation success rate, shortest procedure times, and lowest complication rates when the indication for ERCP was CBDS.

Results of ERCPs for malignancy did not show the same clear pattern as for CBDS. Even if successful cannulation was more common for high-volume endoscopists and centres, procedure times were longer and complication rates, including PEP, were paradoxically higher for endoscopists who performed many ERCPs. ERCP for the diagnosis and treatment of malignancy is often more complicated than ERCP for CBDS, especially if the malignancy is intrahepatic. These procedures are associated with greater risk and higher adverse event rates. ERCP for malignancy is graded at least H.O.U.S.E. II, Schutz IV or Cotton III [3–5]. The paradoxal results of ERCPs performed for malignancy by more experienced endoscopists, with longer procedure times and higher complication rates, may be explained by selection bias. In general, the most experienced high-volume endoscopist performs the most complex and time-consuming ERCP procedures that have the greatest risks for adverse events. Furthermore, high-volume endoscopists use more advanced ERCP techniques such as needle-knife sphincterotomy, and are more likely to persevere longer and spend greater effort cannulating the bile duct before giving up [33].

A limitation of this study is the accuracy of registration of data. Registration of incorrect indication and incompleteness and low frequency of 30-day follow-up affect results and outcome. Regarding complicated ERCP procedures, postoperative complication rate has been shown to be higher in units with a more meticulous follow-up [34]. As yet, GallRiks has not been linked to the Swedish National Patient Register (NPR), so some complications, particularly those occurring after 30 days, may have been missed. However, it is more likely that most adverse events following ERCP occur in the immediate postoperative period.

Unfortunately, ERCP complexity and anatomical differences in the periampullary region are not registered in GallRiks. Administration of indomethacin has been included as a parameter in the quality register the last years but during the period of the study it was not. The parameter previous history of pancreatitis was added to GallRiks very recently (only 6 months ago). Regarding sphincterotomy technique this may differ between high- and low-volume endoscopists, for example more experienced endoscopists tend to use needle knife techniques more frequent [33].

Case-volume is an important issue in ERCP-training, and it is important that the training of future advanced endoscopists is carried out at high-volume center-volume centres. The learning curve among trainees in advanced endoscopy varies significantly. The success rates of trainees performing ERCP, however, increase with increasing experience [35, 36].

This study suggests that greater endoscopist experience and higher centre case-volume are associated with safer and more successful ERCP performance. Acquired experience has a great impact on ERCP outcome for the endoscopist, especially when performed for CBDS. The pattern was not so clear for procedures performed for suspected malignancy. At the centre level, annual volume was similarly associated with better outcome.
